# Digital Innovation in Oncological Primary Treatment for Well-Being of Patients: Psychological Caring as Prompt for Enhancing Quality of Life

**DOI:** 10.3390/curroncol28040224

**Published:** 2021-07-02

**Authors:** Dina Di Giacomo, Federica Guerra, Katia Cannita, Anna Di Profio, Jessica Ranieri

**Affiliations:** 1 Laboratory of Clinical Psychology and Psychoncology, Department of Mesva, University of L’Aquila, 67010 L’Aquila, Italy; federica.guerra@graduate.univaq.it (F.G.); jessica.ranieri@univaq.it (J.R.); 2 Oncology Division, Mazzini Hospital, ASL Teramo, 64100 Teramo, Italy; katia.cannita@gmail.com; 3 Clinical Oncology Division, S.S. Annunziata Hospital, ASL 2 Chieti, 66100 Chieti, Italy; annadiprofio@gmail.com

**Keywords:** chemotherapy, quality of life, well-being, scalp cooling system, oncological patients, psychological treatment, clinical psychology

## Abstract

One side-effect of oncological treatment is chemotherapy-induced alopecia (CIA), a temporary form of hair loss that could influence patients’ mental health. Digitised scalp cooling systems are assuming an important role in the clinical setting during adjuvant treatment, promising hair loss prevention and allowing an efficient procedure to reinforce patients’ mental health during chemotherapy by avoiding CIA. The present study was carried out through two research protocols: in Research Protocol 1, we conducted a randomised clinical study to evaluate the emotional impact of using scalp cooling technology in women with BC compared with a traditional chemotherapy setting; in Research Protocol 2, we conducted an observational pre-post study involving women with BC diagnosis being under adjuvant chemotherapy in two experimental conditions: no scalp cooling application and scalp cooling application. Seventy-four women undergoing chemotherapy, aged 30–55 years, were enrolled in both research protocols. We investigated oncological patients’ psychological dimensions including body image, body appreciation, expectations, and satisfaction with the scalp cooling treatment, with reference to chemotherapy treatment applying the scalp cooling solution. Our data evidenced the need to implement a supportive clinical approach via brief, tailored psychological intervention addressing patients’ progressive adaptation to chemotherapy adverse events and their concerns regarding induced alopecia and the value of the scalp cooling system. Patients receiving the innovative chemotherapy probably coped with it by neglecting its physical impact, instead focusing on avoiding alopecia by using the technological solution and neglecting the emotional impact of chemotherapy as a severe pharmacological treatment.

## 1. Introduction

In primary cancer treatment, patients need to deal with chemotherapy and its associated burdensome physical effects, including effects on body image [1,2,3]. One such side-effect is chemotherapy-induced alopecia (CIA), a temporary form of hair loss that could influence the mental health of patients who suffer from it. In order to support patients dealing with the side-effects of adjuvant treatments, the scalp hypothermia technique was introduced as a preventive measure against the development of CIA. Digitised scalp cooling systems are assuming an important role in the clinical setting during adjuvant treatment, promising hair loss prevention and allowing an efficient procedure to reinforce patients’ mental health during chemotherapy >by avoiding CIA [4,5]. Scalp cooling technology comprises of a refrigerator unit and a control unit integrated into a mobile cabinet and connected to a tight-fitting cooling cap [6,7]. The increasing application of scalp cooling systems in chemotherapy has led to the emergence of an innovative solution in clinical medicine related to mental health during oncological treatments. CIA represents a common and distressing adverse effect of many types of chemotherapy; generally, the onset of CIA occurs two to four weeks after the initiation of chemotherapy. Hair loss is often one of the most feared complications of chemotherapy and is associated with increased psychosocial stress with respect to body image, self-esteem, and sexuality [8,9,10,11]. This innovative preventive system is progressively going to be applied as an evidence-based practice focused on the prevention of induced hair loss [12]. The impact of the scalp cooling device on the well-being and quality of life (QoL) of patients undergoing chemotherapy needs to be investigated by analysing psychological dimensions and mechanisms in depth. Lacking specific literature about the efficacy of scalp cooling system adoption, its use as preventive care for patients’ well-being through the prevention of their hair loss is becoming an emerging topic [13]. Researchers have increasingly investigated the efficacy of scalp cooling technology to measure physical preservation, consider the rate of hair loss, and measure the degree of satisfaction. Innovative solutions should be better studied by considering their mental health-related effects during pharmacological treatment and post-treatment.

This study aimed to investigate the emotional impact of the digital scalp cooling solution for the prevention of chemotherapy-related hair loss. The primary endpoint was to identify the emotional condition of patients dealing with this side-effect of adjuvant treatment when receiving the traditional treatment, compared to the reduced alopecia related to the use of the scalp cooling technology; the secondary endpoint was to analyse the emotional dimensions of patients in chemotherapy, combined with the scalp cooling treatment, identifying the emotional impact of stressors for physical adaptation despite the preserved hair loss: we wanted to study the mental mechanisms dealing with intensive pharmacological treatment using digital solution to reduce side-effects.

We hypothesised that the scalp cooling system, while promising for CIA prevention, may not be considered an elective solution for the quality-of-life protection of patients. To this end, we aimed to test the integrated patient/technology approach in chemotherapy treatment as a means for empowering psychological health outcomes.

## 2. Method

### 2.1. Ethical Approval

This study was approved by the Internal Review Board of the University of L’Aquila, Italy (Prot. No. 55738/2019). Written informed consent was obtained following the Helsinki Declaration (WMA).

### 2.2. Study Design

**Figure 1:**
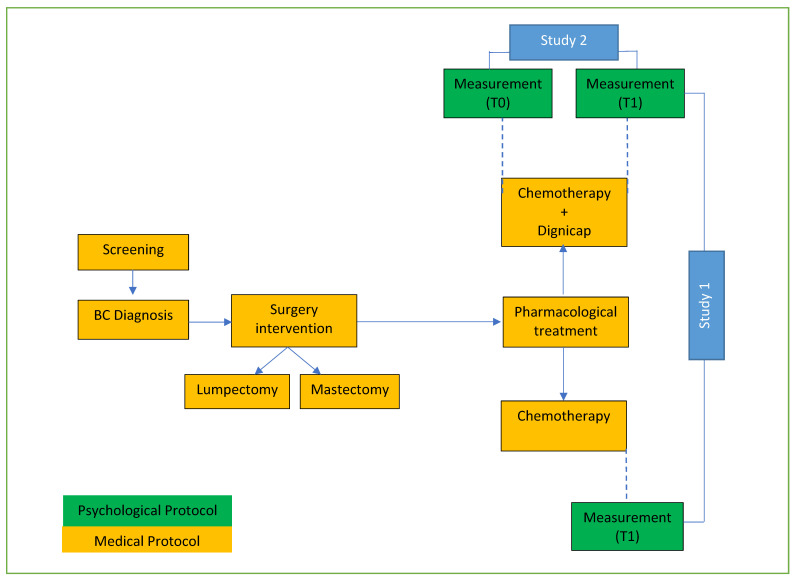
Study Protocol.

The present study was carried out via two research protocols. Figure 1 represents the study design.

In Research Protocol 1, we conducted a randomised clinical control (RCT) study to evaluate the emotional impact of using scalp cooling technology in women with BC. The aim was to evaluate their psychological state after adjuvant chemotherapy in two conditions: Scalp cooling application and no scalp cooling application. The detection data were focused on the emotional impact of chemotherapy at the end of pharmacological treatment, comparing the two clinical settings: traditional setting vs. innovative setting.

In Research Protocol 2, we conducted an observational study based on pre-post psychological measurements involving women diagnosed with BC after being under adjuvant chemotherapy in two experimental conditions: No scalp cooling application and scalp cooling application. All women were given psychological evaluations in pre-post condition, and emotional dimensions such as well-being expectations were detected.

The sample receiving the innovative chemotherapy (pharmacological treatment joined to scalp cooling technology) was the same for both research protocols.

## 3. Research Protocol 1

### 3.1. Objective

The aim of this protocol was to analyse the impact of the scalp cooling technology applied to adjuvant treatment (chemotherapy), evaluating the influence on the emotional outcomes for patients following the end of chemotherapy. An observational study was conducted involving women receiving adjuvant treatment for first time.

### 3.2. Participants

Participants were 44 women aged 30–55 years (mean age 43.85 SD ± 6.45) and diagnosed with BC in TNM cancer stage I-III, who had lumpectomy surgery and were living in Middle Italy. Exclusion criteria were: (a) recurrent or metastatic cancer, (b) premorbid depression and/or anxiety, (c) alcohol or substance abuse. The participants were recruited in ASL1 Abruzzo Healthcare system. Participants were randomised into two groups: the experimental group (digital scalp cooling group; DG), composed of 22 patients undergoing chemotherapy using the scalp cooling treatment, and the control group (no digital scalp cooling group; NDG), composed of 22 patients who received chemotherapy but did not receive the scalp cooling treatment. Patients attended Breast Units from two hospitals of the same Public Healthcare, one of which was provided with the scalp cooling solution for adjuvant treatment in chemotherapy. Table 1 shows demographic data of participants.

**Figure 2:**
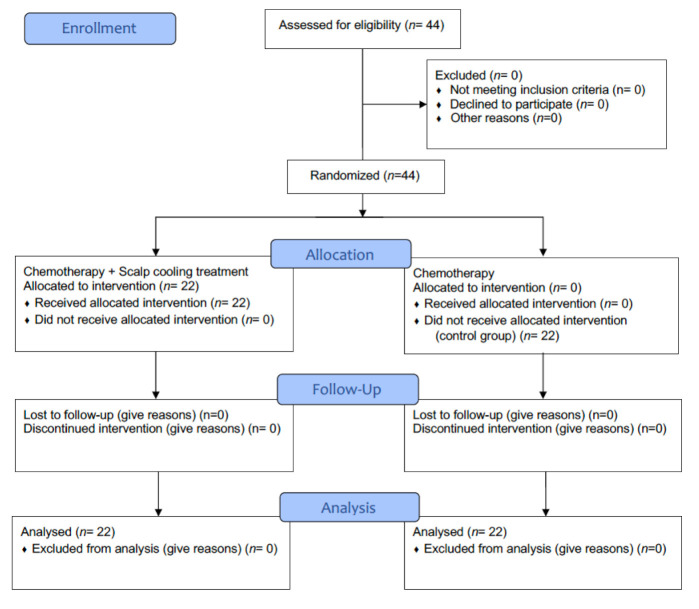
CONSORT Flow diagram for Research Protocol 1.

In Figure 2, the procedure is represented by CONSORT 2010 flow diagram.

### 3.3. Scalp Cooling Technology Treatment

Scalp cooling is performed with a scalp cooling system: a digitised treatment for CIA prevention. The application of this digital solution in medical treatments is preliminarily based on experimental data based on the rate of hair loss.

The system allows a gradual reduction from room temperature to the desired temperature. Deviations from the set temperature are detected and adjusted by the system. The scalp temperature can be controlled with an accuracy of ±2 °C. Scalp cooling begins 30 min before the administration of chemotherapy and is maintained at 5 °C throughout the session. Scalp cooling is applied for each session of chemotherapy, lasting throughout the pharmacological treatment.

### 3.4. Measurement

The psychological battery was composed of two tests, measuring the depression degree (Beck-II) and the psychological distress (PDI). Demographics were collected through participant self-reports; we selected independent variables to be included in the analyses if they fulfilled age/stage of life characteristics (e.g., having children, being employed, and marital status) related to cancer. Second, clinical data were obtained from participants’ medical records regarding BC stage, treatments, and therapies.

*Beck Depression Inventory-II* (BDI-II) [14]. This 21-item self-administered test assesses the intensity of depression in clinical and non-clinical populations. Each item is a list of four statements arranged in increasing severity about a particular symptom of depression. The scores indicate the presence/absence and related degree of depression (from minimal to severe signs of depression). The internal reliability was good for both the DG (α = 0.81) and NDG (α = 0.76) groups.

*Psychological Distress Inventory* (PDI) [15]. This self-administrated questionnaire that measures the impact of psychological distress and related therapies. It is composed of 13 questions, and responses are indicated on a five-point Likert-type scale. The standard score estimates the presence/absence of psychological distress to measure global distress. This test was administered only to the DG group. The inventory demonstrated good reliability (α = 0.86).

### 3.5. Procedure

The medical staff of the Breast Cancer Units identified eligible patients, who were then enrolled during pharmacological treatment (chemotherapy). Informed consent was obtained at the time of enrolment. Patients allocated to the DG group were recruited at the Breast Unit of S. Salvatore Hospital of L’Aquila (Abruzzo, Italy) and provided with scalp cooling devices, whereas the NDG group was recruited from another Breast Unit of the same Healthcare Regional Administration, but not provided with scalp cooling devices. The psychological evaluation was conducted just after the end of chemotherapy treatment. Trained clinical psychologists, blinded from the study’s objectives, conducted the psychological assessments in a quiet, dedicated room. The evaluations lasted about 20 min. The data were collected anonymously.

### 3.6. Statistical Analysis

Descriptive statistics were calculated for T1 measurement in post-pharmacological treatment (see Figure 1). Statistical analyses were performed using Jamovi Stats. One-way ANOVA analyses were performed to verify the statistical significance of the overall differences between the psychological variables after chemotherapy treatment in the DG and NDG conditions. The level of significance adopted was α < 0.05.

### 3.7. Results

**Figure 3:**
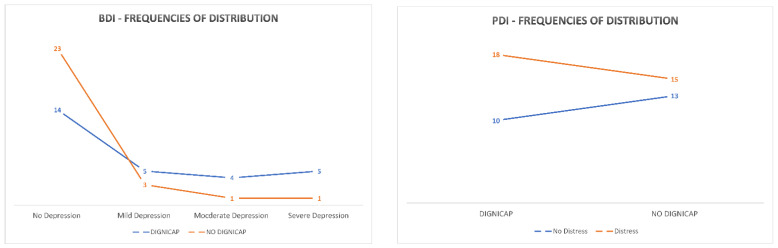
Frequencies of distribution of DG and NDG patients in BDI test.

Distribution data evidenced that most patients showed no sign of depression, whereas low presence of depression signs were seen in both groups. In the DG group, three patients showed mild depression, two showed moderate depression, and three showed severe depression; in the NDG group, two patients reported mild depression, one moderate, and one showed severe depression. Regarding the psychological distress evaluation, more patients showed higher levels of distress (DG, *n* = 12; NDG, *n* = 9) than no distress frequencies (DG, *n* = 10; NDG, *n* = 13). Figure 3 shows the frequencies of distribution of patients in DG and NDG grouping for BDI and PDI testing.

**Figure 4:**
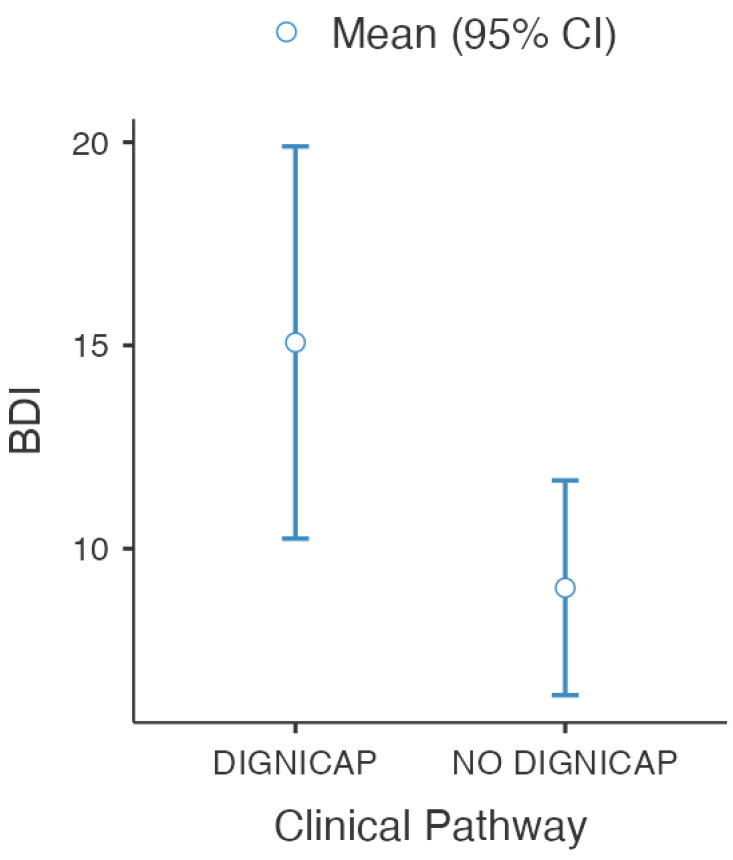
Descriptive Plot for BDI scoring in Post Chemotherapy evaluation.

One-way ANOVA was performed comparing the PDI and BDI total scores for the DG and NDG groups. Statistical analysis showed a significant difference in BDI score (F(1,28) = 41.8; η^2^ = 0.08; *p* = 0.02), evidencing the higher depression risk in the DG group, as shown in Table 2 and Figure 4.

## 4. Research Protocol 2

### 4.1. Objective

The aim of this study was to investigate the dynamics of mental health for patients exposed to chemotherapy joined with the scalp cooling treatment, to identify the emotional impact of stressors for physical adaptation, even with hair loss prevention. We wanted to study the mental mechanisms of dealing with intensive pharmacological treatment combined with the digital solution in preventing CIA. 

### 4.2. Participants

The eligible participants were 30 women aged 29–55 years (mean age 44.9, SD ± 6.2), had lumpectomy surgery, were undergoing chemotherapy using the scalp-cooling treatment, living in Middle Italy, and were diagnosed with BC in TNM cancer stage I-III. Exclusion criteria were: (a) recurrent or metastatic cancer, (b) premorbid depression and/or anxiety, and (c) alcohol or substance abuse. The participants were recruited at the S. Salvatore Hospital—Medical Oncology Division (ASL1) of Abruzzo Healthcare system (Italy). Participants recruited for the observational study with pre-post-chemotherapy psychological testing were *n* = 22; eight women did not take part in the study and, for the following reasons, they did not release their signed informed consents (a—did not time; b—not interested to take part to the study). Table 3 describes the participants’ demographic data.

### 4.3. Measurements

Demographics were collected through participant self-reports; we selected independent variables to be included in the analyses if they fulfilled age/stage of life characteristics (e.g., having children, being employed, and marital status) related to cancer. Second, clinical data were obtained from the participants’ medical records regarding BC stage, treatments, and therapies.

The experimental psychological battery was composed of standardised tests evaluating emotional variables and anxiety (Depression Anxiety Stress Scale 21, DASS-21 only anxiety set), psychological distress (Psychological Distress Inventory, PDI), depression (Beck Depression Inventory 2, BDI-II), alopecia distress (Chemotherapy-Induced Alopecia Distress Scale Italian Version, I-CADS), body image (Body Images Scale, BIS), dependence on body image (Measure of Body Apperception, MBA), and the expectations for and, satisfaction with, using scalp cooling technology during chemotherapy treatment (Expectations And Satisfaction For Scalp cooling Questionnaire, ESDq).

*Psychological Distress Inventory* (PDI) [15]. This self-administrated questionnaire measures the impact of psychological distress and related therapies. It is composed of 13 questions, and responses are indicated on a five-point Likert-type scale. The standard score estimates the presence/absence of psychological distress to measure global distress. This test was administered only to the DG group. The inventory demonstrated good reliability (α = 0.86).

*Beck Depression Inventory-II* (BDI-II) [14]. This 21-item self-administered test assesses the intensity of depression in clinical and non-clinical populations. Each item is a list of four statements arranged in increasing severity about a particular symptom of depression. The scores indicate the presence/absence and related degree of depression (from minimal to severe depression signs). The internal reliability was good for both the DG (α = 0.81) and NDG (α = 0.76) groups.

*Depression Anxiety Stress Scales 21* (DASS-21) [16]. The DASS-21 is a self-administered questionnaire that measures the degree of severity of the core symptoms of depression, anxiety, and stress. It is composed of 21 questions with responses on a four-point Likert-type scale.

*Chemotherapy-Induced Alopecia Distress Scale Italian Version* (I-CADS) [17]. The I-CADS is a self-report test that measures alopecia distress secondary to chemotherapy. The I-CADS consists of 16 items and, specifically, measures self-perception (SP), emotionality (E), and social engagement (SE).

*Body Image Scale, Italian Version* (BIS) [18]. The BIS is a questionnaire that measures the different dimensions of the body image in cancer patients. It consists of 10 items and uses a 4-point response scale (0 = not at all to 3 = a lot).

*Measure of Body Apperception* (MBA) [19]. The MBA is a self-report test that measures investment in, or dependence on, one’s body image as a source of self-worth. It consists of 10 items distributed in two scales, which reflect (a) dependence on physical appearance index (PA) and (b) reliance on a sense of body integrity index (BI).

*Expectations And Satisfaction for Digital Scalp cooling Questionnaire* (ESDq). The ESDq is an ad hoc questionnaire divided into two parts, and measures expectations of, and satisfaction with, the use of scalp cooling technology in conjunction with the chemotherapy treatment, before the start and at the end of using the preventive treatment in conjunction with chemotherapy. The questionnaire consists of 10 items, with 5 items in Part I and 5 items in Part II. Part I is administered before the start of chemotherapy, and Part II at the end of clinical treatment.

### 4.4. Procedure

The medical staff of the Breast Cancer Units identified eligible patients, who were then enrolled before starting pharmacological treatment (chemotherapy). Patients were exposed to pre-chemotherapy (T0) and post-chemotherapy (T1) psychological evaluations. The psychological performance of the sample was evaluated by test-retest procedure. The sample was evaluated at two time-points: before beginning, and at the end of, the chemotherapy treatment, and the same measuring tasks were used at each time point.

Informed consent was obtained at the time of enrolment. Patients recruited at the Breast Unit of S. Salvatore Hospital of L’Aquila (Abruzzo, Italy) were provided with scalp cooling devices. Trained clinical psychologists, blinded from the study’s objectives, conducted the psychological assessments in a quiet, dedicated room. The evaluations lasted about 20 min. The data were collected anonymously.

### 4.5. Statistical Analysis

Research Protocol 2 is an observational study design based on test-retest measurements on the same sample. Descriptive statistics for baseline characteristics and outcome measures at each time point were calculated at T0 and T1 for the DG group (see Figure 1).

Statistical analyses were performed using Jamovi Stats. One-way ANOVA and *t*-test were performed to verify the statistical significance of the overall differences between the psychological variables after chemotherapy treatment in the DG and NDG groups. The level of significance adopted was α < 0.05.

### 4.6. Results

**Figure 5:**
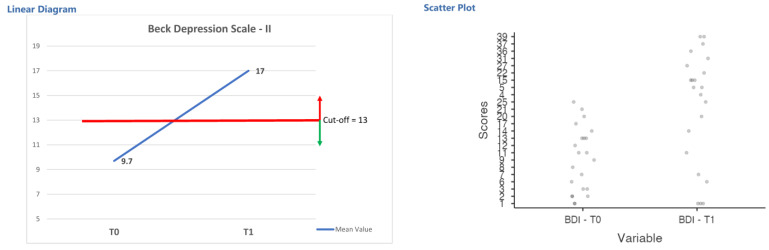
Diagram of test-retest evaluation and scatter plot of depression measure for Research Protocol 2.

Table 4 indicates the raw score (and standard deviation) of the sample in psychological measurements in the test (T0) and retest (T1) procedure. The variables examined were: psychological distress (PDI), depression (BDI), anxiety (DASS-A), body apperception (MBA), body image (BIS), and expectations and satisfaction in using scalp cooling technology (ESDq). The repeated measures ANOVA highlighted significant differences in signs of depression (F(21,1) = 0.02; η^2^ = 0.11; *p* = 0.001); patients showed an increase of signs of mild depression after chemotherapy joined to the scalp cooling solution (see Figure 5).

The comparison test-retest (T0 vs. T1) for other evaluated psychological variables was not significant.

Then, we analysed the alopecia distress (I-CADS) applied at the end of chemotherapy treatment; t-Student analysis showed significant differences in I-CADS total score and in all three related indexes: emotionality, social engagement, and self-perception (see Table 5). A Shapiro–Wilk analysis test evidenced a significant effect of normality distribution for the emotionality index (W = 0.85; *p* = 0.008) and social engagement (W = 0.86; *p* = 0.006). There was no significant difference in normality distribution for self-perception index.

Finally, we examined the correlation between expectations and feelings of satisfaction for the scalp cooling treatment (ESD) and the emotional dimension of the sample after chemotherapy treatment joined with the treatment. Pearson correlation evidenced the expectation and satisfaction feeling of patients who received the scalp cooling solution was negatively correlated to body apperception (MBA) (*p* = 0.004); in particular, the dependence on the physical appearance index was strong (*p* = 0.006), as well the reliance on a sense of body integrity (*p* = 0.04). Moreover, ESD was negatively correlated to alopecia distress (I-CADS; *p* = 0.02), especially the self-perception index (*p* = 0.01). The anxiety measure (DASS-A) influenced the reliance on a sense of body integrity (MBA; *p* = 0.02) as well as the I-CADS self-perception index (*p* = 0.02). Depression degree influenced the psychological distress (*p* = 0.001), the I-CADS self-perception (*p* = 0.002), and emotionality (*p* = 0.004). Psychological distress was correlated positively to MBA indexes (*p* = 0.002), the I-CADS self-perception (*p* = 0.001), and emotionality (*p* = 0.001). Finally, the MBA measure was correlated positively to alopecia distress indexes (*p* = 0.01). Table 6 shows the Pearson’s correlation matrix.

## 5. Discussion

The aim of this study was to investigate the emotional impact of combining the scalp cooling treatment with chemotherapy, analysing the patients’ well-being. The scope of the study was to detect how to better improve the intensive oncological treatments preserving patients’ QoL. Psychological dimensions were investigated, including body image, body appreciation, expectations, and satisfaction with the scalp cooling treatment when combined with chemotherapy. The study was based on two research protocols.

The first research protocol indicated higher negative emotional outcomes after receiving the innovative chemotherapy: women who underwent chemotherapy combined with the scalp cooling solution showed high levels of depression. This point is relevant: innovative solution is promising for CIA prevention, and it could be a buffering effect for psychological distress related to CIA side-effects. Our findings, surprisingly, highlighted that the expectations of patients for physical outcome (no loss of hair) did not modulate the emotional dimensions. The application of the scalp cooling solution could have a positive impact on mental health, and an integrated patient-centred approach treatment should be planned as patients might need psychological support to deal with the effects of pharmacological therapy, pointing out not only the induced alopecia stress, but also body image and physical wellness getting the side-effects of chemotherapy. Van den Hurk et al. [20] suggested that, when scalp cooling does not work as expected, the impact on QoL is worse when compared to controls, i.e., patients not using scalp cooling. As showed in the review by Wang et al. [21], the depression can predict progression of disease: the risk of cancer recurrence is associated with a 17% increase in depression signs, whereas the anxiety is associated with a 13% increase in risk of all-cause mortality. In this perspective, the emerging clinical research needs to address the impact of integrated approaches in primary treatments enhancing the QoL and well-being of patients into empowering care: encouraging the autonomy, self-confidence, and self-determination that facilitates a person’s participation in decision making through effective communication, that is, through the development of an action plan, assisting people to monitor their condition(s) and making changes to their treatment plan.

In our opinion, patients in the control group seemed to be dealing with the side-effect of chemotherapy from early on, adopting mental strategies for future alopecia distress, even choosing how to dress to cope with the alopecia (wig and/or turban). Facing the progression of chemotherapy side-effects, the patients were psychologically focused on their own physical resilience, and that helped them to adapt to the changes over time. Patients receiving the innovative chemotherapy probably dealt with the chemotherapy, neglecting the physical impact as their thoughts were focused on avoiding alopecia, and neglecting the emotional impact of chemotherapy as a severe pharmacological treatment. Oncological primary treatments are intensive and complex but, usually, the fear of induced alopecia represents the primary adverse distressing effect. According to Peterson et al., (2020), patients undergoing the scalp cooling treatment need supportive scalp cooling care to shape patients’ expectations and educate them about efficacy of that innovative care. Peterson at al. [22] suggested integrating the application of the scalp cooling treatment with supportive care by employing physicians and nurses to manage all the adverse effect of oncological treatment, including nausea and vomiting, pain, anaemia, and fatigue. Our data evidence the need to implement a supportive clinical approach using brief and tailored psychological intervention addressed to patients’ progressive adaptation to adverse chemotherapy events and their concerns regarding CIA and the value of the scalp cooling system. The integrated supportive care might improve patients’ psychological resilience to cope with chemotherapy and the scalp cooling treatment. This could be a strategic intervention to favour the most efficient oncological treatments for facilitating a rapid recovery after primary oncological care.

The findings of Research Protocol 2 showed details about the emotional mechanisms underpinning the lower level of well-being after innovative chemotherapy. In particular, results indicated that alopecia distress has a strong influence on the emotional and social engagement dimensions: scalp cooling combined with chemotherapy might mediate the hair loss, but is not decisive since hair loss prevention depends on several factors. Pharmacological regimens (drugs, dose, frequency, number of cycles) and individual factors (hair type) can affect the outcome of scalp cooling efficacy. Moreover, the psychological dimensions of body apperception and physical appearance (concerns about body integrity and appearance) could be affected, depending to the degree of alopecia distress and the level of self-perception; anxiety is involved in this mental process. Lastly, out findings indicated that the signs of depression in the DG group were related to patients’ expectations of scalp cooling efficacy and the impact on their body image and effective hair loss prevention. According to Gianotti et al. [23], patients receiving the scalp cooling treatment for innovative chemotherapy need a tailored intervention for good management of the device, but, in our opinion, it cannot be provided by the nursing team because it is related to emotional dimensions that demand psychotherapy, not just motivational coaching. Based on our findings, we suggest using a multidisciplinary team towards providing empowering care, including mental health professionals to manage the efficacy of complex and intensive adjuvant innovative clinical treatment to prevent, manage, and support the related adverse events of discomfort, fatigue, depression, expectations, and well-being and QoL. Our study highlighted the relevance of psychological intervention to prepare patients for the body image changing before the innovative chemotherapy, focused on the progressive adaptation of patients’ emotions during the intensive pharmacological therapy and acceptance of the psychological phenomena experienced.

## 6. Conclusions

Digital scalp cooling technology is an innovative solution that could improve oncological patients’ QoL because it addresses the side-effects of chemotherapy. However, its efficient application needs to be better exploited, using a multidisciplinary approach involving experts in the clinical care of physical, biological, and psychological aspects of oncological treatment, addressing emotional dimensions and personality mechanisms to facilitate positive outcomes for the patients’ wellness and QoL. This emerging topic needs to be investigated in depth, tailoring interventions into innovative approaches for the implementation of personalised medicine.

## Figures and Tables

**Table 1:** Demographic and clinical data of the sample.

DEMOGRAPHIC INDEXES	POST CHEMOTHERAPY GROUPING		
	DG GROUP (N. 22) x 44.85 sd ± 6.82	NDG GROUP (N.22) x 42.03 sd ± 5.84	Total (N. 44) x 43.85 sd ± 6.45
EDUCATION			
NO HIGH SCHOOL HIGH SCHOOL DEGREE UNDERGRADUATE DEGREE	7.15%	14.28%	10%
	67.85%	57.15%	62%
	25%	28.57%	28%
MARITAL STATUS			
MARRIED/LIVING WITH PARTNER SINGLEDIVORCED/WIDOWED	78.57%	57.14%	67.85%
	3.57%	25%	14.3%
	17.85%	17.85%	17.85%
OCCUPATION			
UNEMPLOYED EMPLOYED SELF EMPLOYED	14.29%	42.85%	28.57%
	60.71%	53.58%	57.15%
	25%	3.57%	14.28%
TNM			
0 1 2 3	3.58%	8%	5.4%
	35.71%	47%	42%
	35.71%	22.5%	29.1%
	25%	22.5%	23.5%
LUMINAL TYPE			
A B HER2 TRIPLE NEGATIVE	10.51%	26%	18%
	7.42%	66%	68%
	7.14%	0%	4%
	10.71%	8%	10%

**Table 2:** Raw score and one-way ANOVA for Research Protocol.

TESTS	POST CHEMOTHERAPY GROUPING		F	*P*
	DG x sd	NDG x sd		
PDI	29.7 (±8.0)	28.1 (±7.4)	0.62	0.43
BDI	15.0 (±12.4)	9.0 (±6.8)	41.8	0.02 *

* *p* < 0.05.

**Table 3:** Demographic data of participants in Research Protocol 2.

DEMOGRAPHIC INDEXES	PARTICIPANTS N(%)
EDUCATION	
NO HIGH SCHOOL	2 (9.1%)
HIGH SCHOOL DEGREE	16 (72.72%)
UNDERGRADUATE DEGREE	4 (18.18%)
MARITAL STATUS	
MARRIED/LIVING WITH PARTNER	17 (77.27%)
SINGLE	1 (4.55%)
DIVORCED/WIDOWED	4 (18.18%)
OCCUPATION	
UNEMPLOYED	1 (4.55%)
EMPLOYED	19 (86.35%)
SELF EMPLOYED	2 (9.1%)
TNM	
0	2 (9%)
1	9 (40.9%)
2	5 (22.77%)
3	6 (27.33%)
LUMINAL TYPE	
A	1 (4.56%)
B	17 (77.27%)
HER2	1 (4.56%)
TRIPLE NEGATIVE	3 (13.63%)

**Table 4:** Raw Score of Psychological measurements and Repeated Measures ANOVA for Research Protocol 2.

TEST	TIMEPOINT	RAW SCORE DG		F	*P*	*H* ^2^
		χ	sd			
PDI	T0	28.2	±7.46	0.74	0.39	-
	T1	29.4	±8.72			
BDI	T0	9.7	±6.98	12.65	0.001 *	0.11
	T1	17.0	±13.13			
ESDQ	T0	16.0	±3.39	1.42	0.24	-
	T1	17.0	±3.13			
MBA (TOTAL)	T0	15.5	±5.19	2.70	0.11	-
	T1	18.0	±4.71			
MBA (PA INDEX)	T0	9.4	±3.23	1.69	0.20	-
	T1	10.0	±2.84			
MBA (BI INDEX)	T0	7.0	±2.80	1.08	0.31	-
	T1	7.9	±2.68			
BIS	T0	9.0	±8.32	4.22	0.05	-
	T1	11.1	±8.10			
DASS-A	T0	9.5	±9.19	0.05	0.81	-
	T1	9.1	±6.80			

* *p* < 0.05.

**Table 5:** I-CADS raw score of sample after chemotherapy treatment and *t*-test one sample.

Tests	χ	sd	*t*	*p*	*Cohen’s d*
I-CADS Self-perception *index*	16.1	±5.72	13.2	0.01	2.82
I-CADS Emotionality *index*	5.5	±2.32	11.82	0.01	2.36
I-CADS Social Engagement *index*	7.2	±3.23	10.4	0.01	2.17
I-cads *total score*	28.8	±9.81	13.7	0.01	2.88

**Table 6:** Correlation Matrix (Pearson’s *r*) of emotional dimensions of sample at T1.

		ESDQ T1	DASS-A T1	BDI - T1	PDI T1	MBA T1	MBA IA T1	MBA IBI T1	I-CADS SP	I-CADS E	I-CADS- SE	I-CADS Tot
ESDQ T1	Pearson's *r*	—										
	*p*-value	—										
DASS-A T1	Pearson's *r*	−0.412	—									
	*p*-value	0.057	—									
BDI - T1	Pearson's *r*	−0.339	0.311	—								
	*p*-value	0.122	0.159	—								
PDI T1	Pearson's *r*	−0.42	0.408	0.613 **	—							
	*p*-value	0.052	0.06	0.002	—							
MBA T1	Pearson's *r*	−0.591 **	0.413	0.235	0.624 **	—						
	*p*-value	0.004	0.056	0.292	0.002	—						
MBA IA T1	Pearson's *r*	−0.568 **	0.23	0.038	0.43 *	0.86 ***	—					
	*p*-value	0.006	0.302	0.867	0.046	<0 .001	—					
MBA IBI T1	Pearson's *r*	−0.435 *	0.48 *	0.372	0.637 **	0.842 ***	0.45 *	—				
	*p*-value	0.043	0.024	0.088	0.001	<0 .001	0.036	—				
I-CADS SP	Pearson's *r*	−0.495 *	0.482 *	0.629 **	0.639 **	0.621 **	0.397	0.669 ***	—			
	*p*-value	0.019	0.023	0.002	0.001	0.002	0.067	< 0.001	—			
I-CADS E	Pearson's *r*	−0.276	0.08	0.585 **	0.79 ***	0.466 *	0.417	0.375	0.566 **	—		
	*p*-value	0.213	0.723	0.004	< 0.001	0.029	0.054	0.086	0.006	—		
I-CADS- SE	Pearson's *r*	−0.369	0.234	0.314	0.418	0.5 *	0.52 *	0.325	0.648 **	0.526 *	—	
	*p*-value	0.091	0.295	0.154	0.053	0.018	0.013	0.139	0.001	0.012	—	
I-CADS Tot	Pearson's *r*	−0.478 *	0.379	0.617 **	0.7 ***	0.652 **	0.512 *	0.6 **	0.938 ***	0.743 ***	0.827 ***	—
	*p*-value	0.024	0.082	0.002	<0 .001	0.001	0.015	0.003	<0 .001	< 0.001	< 0.001	—

Note. * *p* < 0.05, ** *p* < 0.01, *** *p* < 0.001.

## Data Availability

The data presented in this study are available on request from the corresponding author. The data are not publicly available due to the exploration of the study by other ongoing researches.
